# Extensive Nicolau syndrome following intramuscular diclofenac sodium injection^⋆^

**DOI:** 10.1016/j.abd.2022.06.010

**Published:** 2023-09-22

**Authors:** Rafael Oliveira Amorim, Alana Luísa Calixto Carlos da Silva, Camila Arai Seque, Adriana Maria Porro

**Affiliations:** Department of Dermatology, Universidade Federal de São Paulo, São Paulo, SP, Brazil

Dear Editor,

Nicolau syndrome (NS), or embolia cutis medicamentosa, is vascular occlusion with necrosis of the skin and underlying tissues, related to the use of medications such as beta-lactam antimicrobials, non-steroidal anti-inflammatory drugs (NSAIDs) and, more recently, hyaluronic acid fillers.

A 66-year-old male patient reported erythema and ecchymosis on the left lower limb, 24 hours after an intramuscular injection (left gluteus) of diclofenac sodium for abdominal pain. There was rapid progression to tense blisters over the entire limb, with severe pain and edema.

Four days later, the patient came to the emergency room with rectilinear purpuric areas all over the left lower limb, tense blisters with serosanguineous content, and ulcerated areas (Fig. 1A).

**Figure 1:**
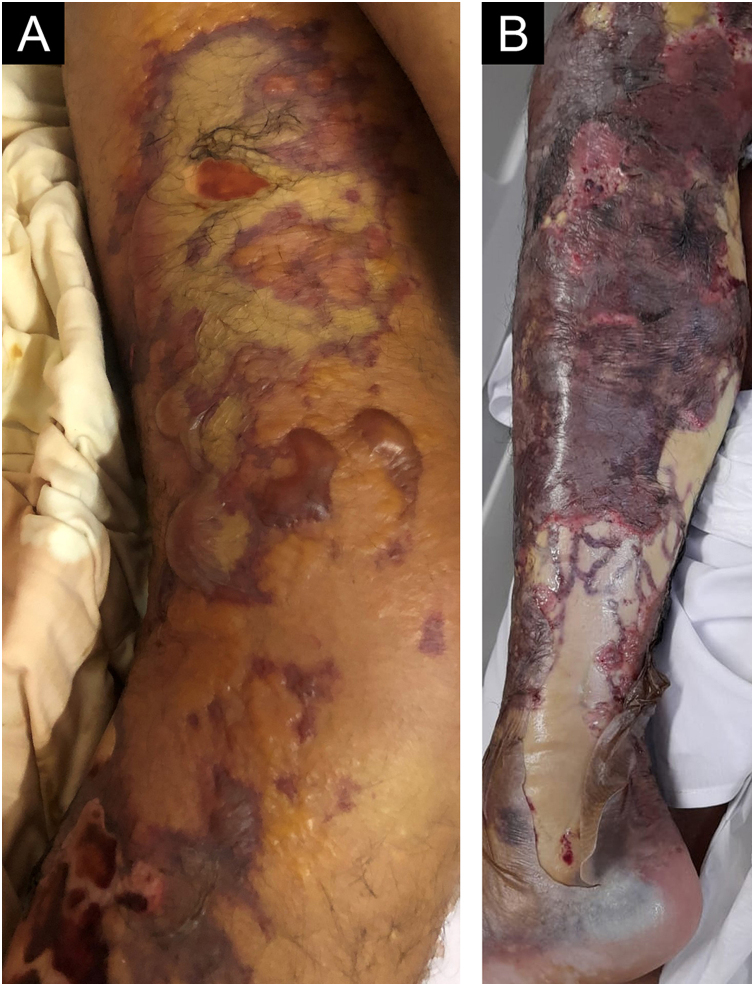
(A) Violaceous, rectilinear lesions with areas of skin detachment on the thigh and leg. Areas of necrosis with an erythematous halo are also observed. (B) Progression of the necrosis areas seven days after symptom onset

The hypothesis of NS due to intramuscular sodium diclofenac injection was considered. Laboratory investigation for systemic vasculitis with antinuclear antibody (ANA), anti-DNA, extractable nuclear antigen (ENA), cryoglobulins and complement was negative.

The lesions worsened (Fig. 1B), and the patient was transferred to the Intensive Care Unit and received parenteral antibiotic therapy (vancomycin and meropenem). After clinical stabilization, surgical debridement sessions were performed (Fig. 2) with subsequent grafting (Fig. 3). After three months and four surgical approaches for debridement and grafting, the patient was discharged in good general condition.

**Figure 2:**
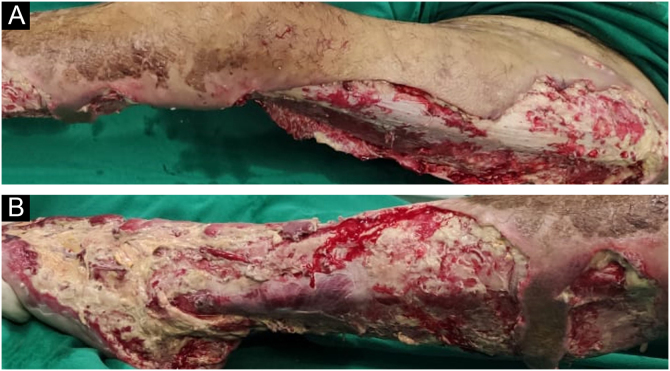
After 30 days, in the postoperative period of the first surgical debridement on the left thigh (A) and left leg (B)

**Figure 3:**
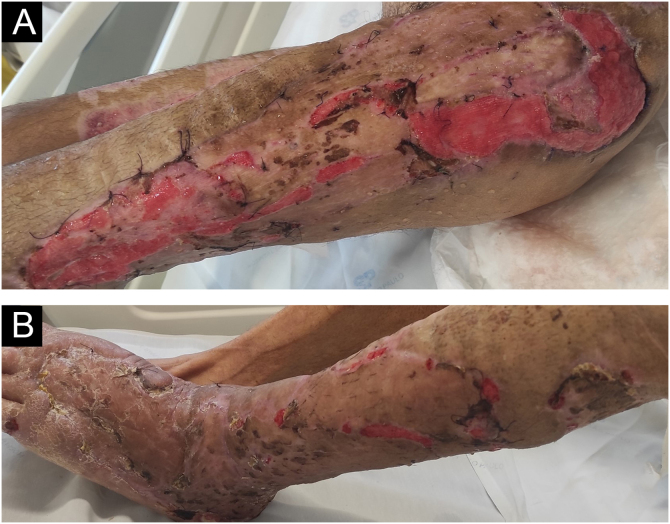
Three months after the onset of the condition, the patient shows good aesthetic and functional recovery in the (A) right thigh and hip and (B) right leg and dorsum of the right foot after multiple skin grafts

NS was first described in 1924 after an intragluteal injection of bismuth salts for the treatment of syphilis. It occurs after the intramuscular injection of insoluble substances, occurring more frequently with benzathine penicillin and NSAIDs (such as diclofenac).^1^ Diclofenac-induced cases are more common in females, whereas penicillin-induced cases are more common in children.^2^

Theories about its etiopathogenesis involve a combination of factors: stimulation of sympathetic innervation with vasospasm and ischemia; blockade of prostaglandin synthesis by NSAIDs, arterial embolic occlusion by inadvertent intravascular injection; perivascular inflammation due to cytotoxic drug reaction; mechanical injury caused by lipophilic drugs penetrating the vessels.^2^, ^3^

The clinical picture consists of an erythematous macule with rapid evolution to a livedoid violaceous patch. The onset is usually sudden in relation to the injection but can be delayed, often without injury to the injected site.^4^ The prognosis is unpredictable, with reports of recovery and atrophic scarring at the affected site but also compartment syndrome, hyperkalemia, renal failure, paralysis of the affected limb, and death.^3^, ^5^ The diagnosis is clinical, highly suggestive when the lesion starts at the injection site, with distal progression over the injected limb. Histopathology is non-specific and may reveal fatty tissue necrosis and inflammation.^5^

There is no specific treatment, and analgesia, treatment of secondary infection, and surgical debridement are employed.^1^ The correct intramuscular injection technique can reduce the risk of the condition.^4^ The Z-track injection method is recommended, with traction of the skin and subcutaneous tissue prior to needle insertion, ensuring blockage of the needle path after the injection.^1^

Although rare, NS can be extensive and severe. Physicians must be aware of the condition, as well as aware of the correct injection technique, and avoid unnecessary intramuscular prescriptions.

## Financial support

None declared.

## Authors' contributions

Rafael Oliveira Amorim: Drafting and editing of the manuscript and critical review of important intellectual content; study design together with the co-authors; critical review of the literature; approval of the final version of the manuscript.

Alana Luísa Calixto Carlos da Silva: Drafting and editing of the manuscript and critical review of important intellectual content; study design together with the co-authors; approval of the final version of the manuscript.

Camila Arai Seque: Drafting and editing of the manuscript and critical review of important intellectual content; study design together with the co-authors; approval of the final version of the manuscript.

Adriana Maria Porro: Drafting and editing of the manuscript and critical review of important intellectual content; study design together with the co-authors; approval of the final version of the manuscript.

## Conflicts of interest

None declared.
